# Evaluation of the Prognostic Value of IFN-γ Release Assay and Tuberculin Skin Test in Household Contacts of Infectious Tuberculosis Cases in Senegal

**DOI:** 10.1371/journal.pone.0010508

**Published:** 2010-05-06

**Authors:** Christian Lienhardt, Katherine Fielding, Abdoul A. Hane, Aliou Niang, Cheikh T. Ndao, Farba Karam, Helen Fletcher, Fatou Mbow, Jules-François Gomis, Roger Diadhiou, Maxime Toupane, Tandakha Dieye, Souleymane Mboup

**Affiliations:** 1 Institut de Recherche pour le Développement, Programme Tuberculose, Dakar, Senegal; 2 Infectious Disease Epidemiology Unit, London School of Hygiene & Tropical Medicine, London, United Kingdom; 3 Service de Pneumologie, Hôpital Fann, Dakar, Senegal; 4 Laboratoire de Bactériologie–Virologie, Hôpital Aristide Le Dantec, Dakar, Senegal; 5 Centre for Clinical Vaccinology and Tropical Medicine (CCVTM), University of Oxford, Churchill Hospital, Oxford, United Kingdom; McGill University, Canada

## Abstract

**Background:**

Chemoprophylaxis of contacts of infectious tuberculosis (TB) cases is recommended for TB control, particularly in endemic countries, but is hampered by the difficulty to diagnose latent TB infection (LTBI), classically assessed through response to the Tuberculin Skin Test (TST). Interferon-gamma release assays (IGRA) are proposed new tools to diagnose LTBI, but there are limited data on their ability to predict the development of active TB disease. To address this, we investigated the response to TST and IGRA in household contacts of infectious TB cases in a TB high-burden country and the potential correlation with development of TB.

**Methodology/Principal Findings:**

Prospective household contacts study conducted in two health centres in Dakar, Senegal. A total of 2679 household contacts of 206 newly detected smear and/or culture positive index TB cases aged 18 years or greater were identified A TST was performed in each contact and an ESAT6/CFP10 ELISPOT assay performed in a random sample of those. Contacts were followed-up for 24 months. TB was diagnosed in 52 contacts, an incidence rate of 9.27/1000 person-years. In univariable analysis, the presence of positive TST (≥10 mm) and ELISPOT (>32 SFC/million PBMC) responses at baseline were associated with active TB during follow-up: Rate Ratio [RR] = 2.32 (95%CI:1.12–4.84) and RR = 2.09 (95%CI:0.83–5.31), respectively. After adjustment for age, sex and proximity to index case, adjusted RRs were 1.51 (95%CI:0.71–3.19) and 1.98 (95%CI:0.77–5.09), respectively. Restricting analysis to the 40 microbiologically confirmed cases, the adjusted RR for positive ELISPOT was 3.61 (95%CI:1.03–12.65). The median ELISPOT response in contacts who developed TB was 5-fold greater than in those who did not develop TB (p = 0.02).

**Conclusions/Significance:**

TST and IGRAs are markers of a contact of the immune system with tubercle bacilli. In a TB endemic area, a high ELISPOT response may reflect increased bacterial replication that may subsequently be associated with development of TB disease and may have a prognostic value. Further longitudinal data are needed to assess whether IGRAs are reliable markers to be used for targeting chemoprophylaxis.

## Introduction

For decades, the diagnosis of Latent Tuberculosis Infection (LTBI) has relied on the Tuberculin Skin Test (TST), which measures a delayed-type hypersensitivity response to a purified protein derivative (PPD) of more than 200 *M. tuberculosis* antigens [1]. TST suffers, however, limitations due to cross-reactions with a wide range of environmental mycobacteria and the BCG vaccine [2]. The characterisation of immunogenic antigens in the Region of Difference 1, a genomic region present in the *M. tuberculosis* complex but deleted from *M. bovis* BCG and most environmental mycobacteria, has allowed the development of highly-specific immuno-diagnostic tests for TB infection [3]. These tests measure the release of interferon-γ by blood T cells that have been activated in-vitro by *M. tuberculosis* specific antigens, mainly ESAT-6 and CFP10 [4]. Studies have suggested that Interferon-γ Release Assays (IGRAs) using the ESAT-6/CFP-10 antigens were more specific than the TST for the diagnosis of latent TB infection [5] and at least as sensitive. Subsequently, IGRAs are increasingly recommended for the detection of LTBI [6], [7]. In addition, based on the hypothesis that T cell response to TB specific antigens correlate with bacterial replication, it has been suggested that IGRAs may help to identify individuals at greatest risk for development of active TB disease who may therefore benefit from preventive therapy [8], [9]. There are however limited data on the prognostic ability of IGRAs to predict the development of active TB disease. We report here results of a prospective household contact study carried out in Senegal, in which contacts of active TB cases were tested at baseline with an in-house ESAT-6/CFP-10 ELISPOT assay (referred to as “ELISPOT”) in the rest of the article and followed-up for two years to detect occurrence of tuberculosis.

## Methods

### Setting

The study was conducted in Dakar, Senegal, from March 2004 to April 2006. The total population in Dakar is 2 million, and the reported incidence of newly diagnosed sputum smear–positive TB is 132 cases per 100,000 population per year [10].

### Design

Newly detected pulmonary tuberculosis cases aged 18 years or greater with at least 1 sputum smear positive for acid-fast bacilli and/or a positive culture, identified in two health centres in Dakar (Fann Hospital and Pikine Health Centre), who were living at the same address for more than 3 months and gave informed consent, were eligible for inclusion in the study. An antero-posterior chest X-ray was performed in each case, and was read by one of the clinicians. After counselling, cases were invited to undergo an HIV test.

The household of each index case was visited by trained field assistants within a week of their recruitment. We defined household as the extended family living together in the same area, eating from the same pot [11]. A written informed consent was obtained from each household member or child care-taker prior to enrolment. Demographic information was collected from all individuals (adults and children) living in the household for more than 3 months as well as their past disease history, presence of risk factors for TB and relatedness to the index case. Intensity of exposure to the index case within the household was estimated through a purposely defined gradient of exposure evaluating the physical proximity of the household member to the index case at night-time: (i) share same room and same bed as the index case; (ii) shares same room but not same bed; (iii) shares same house but not same room; (iv) dos not sleep in the same house [11]. In addition, the amount of time and activities shared with the case during the day were estimated as: “eating with the index case (i) daily, (ii) less than daily, (iii) not eating with the case; as well as”recreational activities with the index case (i) daily, (ii) less than daily, (iii) not at all. Following national guidelines, mothers/guardians of children ≤5 years old were advised to go the health centre for a 6-month prophylaxis with isoniazid, and a special transfer voucher was delivered to them for this.

Each household member was examined for the presence of a BCG scar, and screened for signs and symptoms of tuberculosis. Those in whom TB was diagnosed as a result of the enrolment visit were excluded from all analyses. Blood samples were obtained for ELISPOT from 10 randomly selected contacts per household: all members were listed and their name written on pieces of paper that were put in an urn. A person was chosen to pull out names of 10 household members who underwent ELISPOT testing. If the household size was <10, then all members underwent ELISPOT testing. All contacts were skin-tested with tuberculin (RT23 2TU, Staten Serum Institute) on the same day, after blood was drawn. Trained field workers visited all contacts after 48–72 hours to measure the skin induration.

Households were visited at 3, 6, 12, 18 and 24 months after recruitment into the study, in order to identify persons who left the household (either temporarily or permanently) and detect any suspect TB case. Household members were educated on the signs and symptoms of the disease to encourage self-referral to Fann Hospital in the event of suspected tuberculosis. At each home visit, household members were queried on symptoms and signs suggestive of TB (cough >3 weeks, fever, loss of weight, night sweats, chest pain, haemoptysis). Suspect TB cases were referred to the same clinician at Fann Hospital for clinical examination and further investigation, including collection of two sputa for smear and culture, chest X-ray and gastric lavage. Chest X-rays were read independently of TST and IGRAs results. In order to account for the difficulty of diagnosing tuberculosis in adults with smear negative or extra-pulmonary disease and in children, we set up a certainty grading system that quantifies the likelihood of tuberculosis diagnosis, based on several published systems [11]. Cases of tuberculosis were graded as “possible”, “probable” or “definite” (see Box S1).

#### The ESAT-6/CFP-10 IFN-γ ELISPOT assay

The assay was performed on freshly isolated peripheral blood mononuclear cells (PBMCs), as previously described [12], predominantly using one pool of 35 15-mer peptides, overlapping by 10 amino-acids (10 µg/ml) and spanning the length of CFP-10 (18 peptides) and ESAT-6 (17 peptides) (Mabtecch AB, Sweden). Briefly, 200,000 PBMCs per well were plated directly onto the ELISPOT plate (MAIP, Millipore) in the presence of the peptides, and incubated for 18 hours. Phytohaemagglutinin (PHA) (5 µg/ml) (Sigma, Missouri, USA) as positive control and media as negative control, were added to duplicate wells. ELISPOT plates were counted using an AID plate reader (Autoimmun Diagnostika, Strasburg, Germany).

The in-house EC-ELISPOT assay performed in Senegal is equivalent to the EC-ELISPOT assay performed in The Gambia [13]. The technique originated from the same Oxford University laboratory and a trained laboratory technician from the Gambia helped to establish the assay in Senegal. Head to head comparisons of the Gambian and Senegalese assays were performed and good correlation was found between the two assays (data not shown).

We set two thresholds for positive response to an antigen pool: (i) more than 20 spot forming cells (SFC)/10^6^ PBMC after negative control well SFC subtraction and (ii) more than 32 SFC/10^6^ PBMC after negative control well SFC subtraction. The cut-off of 32 SFC/million was selected as it is equivalent to the cut-off of 8 SFC/well used in previously published EC-ELISPOT studies conducted in The Gambia [13]. The lower cut-off of 20 SFC/million is closer to that of the commercial T-SPOT assay and the original ESAT-6/CFP-10 ELISPOT assay [14]. Assays were excluded if there were more than 20 spots in the negative control wells, and the remaining n = 1196 assays were used for analysis. Assays were considered valid if there were at least 250 SFC/10^6^ PBMC in either PHA or purified protein derivative (PPD) wells. Amongst the 1196 assays, n = 952 (80%) had a valid PHA/PPD response and n = 244 (20%) assays were considered invalid and therefore excluded from further analysis.

### Analysis

Risk factors for time to TB episode during follow-up were analysed using Cox proportional hazards regression. The primary risk factors of interest were TST and ELISPOT responses at enrolment. Other factors measured at the household or TB index level were considered potential confounders or effect modifiers. The period of risk started at the date of household member enrolment plus 60 days so as to exclude co-prevalent TB cases (ie. TB cases in household members diagnosed as a result of the enrolment visit). The period of risk ended at either the 24 month follow-up visit, the midpoint of the last visit seen (if earlier than month 24) and the following scheduled visit date, date of death or date of TB diagnosis (defined as the start date for treatment) whichever came first. Factors with a P value of <0.10 or those that changed the hazard ratio for TST or ELISPOT response by greater than 10% were considered for the multivariable analysis. Age group and gender were considered *a priori* confounders. Variables were retained in the model if their inclusion changed the effect of the TST or ELISPOT response on TB incidence by greater than 10%. The likelihood ratio test was used to assess general association between risk factors and outcome. Tests for departures from linearity and for linear trend were carried out for the association of ordinal factors with outcome.

### Ethical aspects

This study proposal was approved by the Ethics Review Committee of the European Commission. The full protocol and informed consent forms were approved by the Ethical Committee of the National Centre for Scientific Research of the Ministry of Health, Senegal.

International guidelines recommend that children ≤5 years who are contacts of a smear positive TB case and are TST positive be offered preventive therapy with isoniazid (IPT) at a dose of 5 mg/kg for 6 months. The Senegalese ethics committee recommended that we follow these guidelines. Subsequently, at recruitment, parents/guardians of child contacts aged ≤5 years were requested to go to the local health clinic to get preventive therapy and a special referral form was completed and given to them for this. Unfortunately, the provision of IPT to children was very irregularly ensured at the public health centres in Senegal at the time of the study. Since anti-tuberculous treatment in Senegal can only be provided through the National TB Control Programme, the study team was unable to provide IPT to all child contacts recruited in the study.

## Results

**Figure 1:**
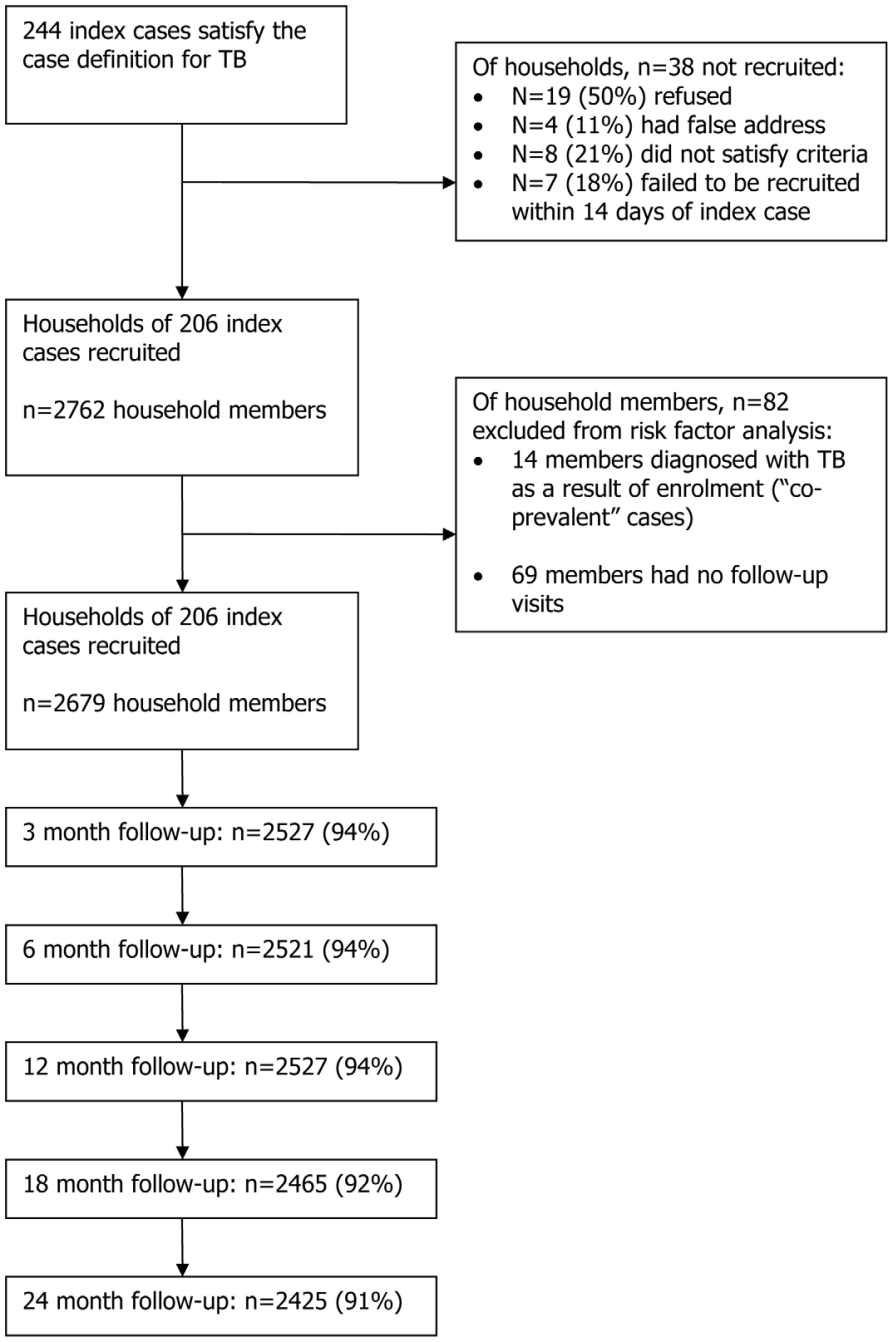
Study Profile.

From March 2004 to April 2006, 244 smear positive index TB cases were recruited. The households of 206 (84%) of these cases were included in the study, representing 2762 household members. The reasons for 38 households of index cases not being included in the study are summarised in **Figure 1**. Of the 2762 household members, 13 (0.5%) were diagnosed with TB as a result of screening at the enrolment visit, and one died with TB within 60 days of recruitment. A further 69 (2.5%) household members had no follow-up visits, leaving 2679 contact persons from 206 households available for analysis (**Figure 1**). The median age of the 206 index cases was 28 years (range 18–71 years), 68% were male and the median time with cough prior to TB diagnosis was 6 weeks (range 1–52 weeks) (**Table 1**). All were smear positive and 85% were culture positive. The large majority (85%) had at least one cavity on the chest X-ray.

**Table 1:** Summary of index cases (n = 206).

Variable	Coding	Summary	
Age (years)	Median (IQR)Range	28 (23, 38)18, 71	
Gender^1^ n (%)	MaleFemale	*n*13966	*(col %)*(67.8)(32.2)
Culture^2^	PositiveNegative	10619	(84.8)(15.2)
Smear positivity^3^	Scanty^4^1+2+3+	8456978	(4.0)(22.5)(34.5)(39.0)
Cavitation on chest radiograph^5^	01–23–8	2710353	(14.7)(56.3)(29.0)
Number of zones on chest radiograph^6^	0–34–56	150417	(75.8)(20.7)(3.5)
Cough duration^7^ (weeks)	<33–45–8>8	18715750	(9.2)(36.2)(29.1)(25.2)
HIV status^8^	PositiveNegative	567	(4.3)(95.7)

1 n = 1 missing;

2 n = 81 missing;

3 degree of positivity not known for n = 6 (all known to be smear positive);

4 of n = 8 scanty, n = 1 had a single smear scanty and culture positive, n = 4 have two scanty smears and n = 3 have three scanty smears;

5 n = 23 missing;

6 n = 8 missing;

7 n = 10 missing;

8 n = 134 missing.

IQR =  interquartile range; col = column

Of the 2679 household members, 46% were female and the median age was 20 years (interquartile range [IQR] 10–31 years), including 390 (14.6%) children aged ≤5 yrs. The median household size was 12 persons (IQR: 7–17), and the large majority of members (86%) had lived in the household for more than 12 months. TST was performed and measured on 2458 individuals, while ELISPOT response was measured in 952 household members (n = 186 households) only, due to financial constraints.

Follow-up of household members at the scheduled visits is summarised in **Figure 1**. At month 12 and 24, 94% (n = 2527) and 91% (n = 2425) were present at the follow-up visit, respectively. The median follow-up was 1.91 years (IQR: 1.83–2.59) giving a total of 5606.5 person-years [pyrs] of follow-up. During this time, 52 cases (2%) satisfying the case definition for TB were diagnosed, giving an incidence rate of 9.27/1000 pyrs (95% confidence interval [CI]: 7.07–12.17) (**Table 2**). The 52 secondary cases came from 41 households, with 32 households having one secondary case, 8 households having two secondary cases and one household having four secondary cases. The median age was 25 years (IQR 18.5–32), and 56% were females. The median time to development of TB was 8.3 months (range 0.4–28.4 months). The large majority of cases (n = 43, 84%) had pulmonary TB, while 7 (13%) had extra-pulmonary disease and 2 (4%) had extra-pulmonary disease associated with pulmonary TB. Forty incident TB cases satisfied the “definite” case definition, based on microbiology results.

**Table 2:** Summary of secondary cases in household members (n = 52).

Variable	Coding	Overall summary(n = 52)		Summaryage <16 yrs(n = 6)		Summaryage ≥16 yrs(n = 46)	
Age (years)	Median (IQR)Range	25 (18.5,32)0, 70		140, 15		2716, 70	
		*n*	*(col %)*	*n*	*(col %)*	*n*	*(col %)*
Gender	MaleFemale	2329	(44.2)(55.8)	24	(33)(67)	2125	(46)(54)
Smear status	Positive	34^1^	(72.3)	4^2^	(80)	30^3^	(71)
Smear positivity (of those smear positive)	Scanty1+2+3+	191410	(2.9)(26.5)(41.2)(29.4)	0202	(50)(50)	17148	(3)(23)(47)(27)
Culture	Positive	28^1^	(59.6)	3^2^	(60)	25^3^	(60)
Definition of TB	DefiniteProbablePossible	40210	(76.9)(3.8)(19.2)	402	(67)(33)	3628	(78)(4)(17)
Type of TB	PTB aloneEPTB alonePTB+EPTB	4372	(83.7)(13.5)(3.8)	501	(83)(17)	3871	(83)(15)(2)
Time to diagnosis of TB (months) 4	Median (IQR)Range	8.3 (3.3–11.9)0.4, 28.4		6.41.1–14.8		8.3 (3.5 – 12.3)0.4 – 28.4	

1 n = 5 missing,

2 n = 1 missing,

3 n = 4 missing,

4 measured from HH member enrolment +60 days.

IQR = interquartile range; PTB = pulmonary TB; EPTB = extra-pulmonary TB; col = column.

The univariable analysis is presented in **Table 3** for all TB cases (n = 52), and in **Table 4** for definite TB cases only (n = 40). A TST response of 10 mm or more was associated with over a two-fold increase in the risk of TB (Hazard Ratio [HR]: 2.37, 95%CI: 1.15–4.89). When TST response was categorized into four levels, there was nearly five-fold increase in risk for those with a response 15 mm or greater compared with a baseline level of <5 mm (HR: 4.92, 95%CI: 1.74–13.92). Among household members in whom ELISPOT was being tested, those who presented an ELISPOT response ≥32 SFC/10^6^ PBMC had more than a two-fold increased risk of developing TB in follow-up compared with those with a response <32 SFC/10^6^ PBMC (HR: 1.98, 95%CI: 0.74–5.33). When restricting the analysis to definite TB cases only (n = 40) (**Table 4**), the risk of developing TB given a positive TST response increased slightly. The association with a positive ELISPOT response appeared, however, stronger, with a nearly 4-fold increase in hazard of developing TB when response at baseline was ≥32 SFC/10^6^ PBMC.

**Table 3:** Univariable analysis of time to TB episode; hazard ratios, 95% CI and P-values (n_max_ = 2679) - All TB cases (n = 52).

Variable	Description	n (col %)	Rate/1000 pyrs	TB cases/pyrs	Unadjusted HR(95% CI)	P-value
TST response^1^ (mm)	<10≥ 10	867 (35)1591 (65)	4.8911.71	9/184039/3332	12.37 (1.15–4.89)	0.01
TST response 1 (mm)	<55–910–14≥ 15	548 (22)319 (13)688 (28)903 (37)	3.317.274.7516.49	4/11955/6457/144332/1889	12.18 (0.59–8.13)1.40 (0.41–4.79)4.92 (1.74–13.92)	0.0002
EC response^2^(SFC/million PBMC)	<20≥ 20	337 (35)615 (65)	6.8513.56	5/73018/1327	11.98 (0.74–5.33)	0.15
EC response^2^(SFC/million PBMC)	<32≥ 32	408 (43)544 (58)	6.8614.37	6/87517/1183	12.13 (0.84–5.41)	0.09
Gender	MaleFemale	1420 (53)1259 (47)	8.759.74	23/263029/2977	11.11 (0.65–1.93)	0.70
Age (years)	≤1516–29≥ 30	1031 (38)897 (33)751 (28)	2.7115.3011.54	6/221628/183018/1560	15.50 (2.28–13.29)4.18 (1.66–10.52)	<0.0001
Duration of residence with index case^3^ (months)	<66–12>12	199 (7)173 (6)2293 (86)	2.472.9710.33	1/4041/33650/4840	0.23 (0.03–1.67)0.27(0.04–1.96)1	0.05
Eat with index case^4^	NoLess than dailyDaily	745 (28)660 (25)1273 (48)	5.895.1213.28	9/15277/136636/2711	10.87 (0.33–2.35)2.33 (1.12–4.83)	0.007
Proximity to index case at night^4^	Other houseSame houseSame roomSame bed	430 (16)1764 (66)213 (8)271 (10)	7.216.6418.0423.08	6/83225/37668/44413/563	11.00 (0.41–2.43)2.69 (0.93–7.75)3.43 (1.30–9.02)	0.002
Household size (number of people)	<1010–1415–19≥ 20	461 (17)700 (26)568 (21)950 (35)	9.025.3916.838.02	9/9988/148419/112916/1995	10.59 (0.23–1.52)1.75 (0.79–3.86)0.86 (0.38–1.95)	0.04
Previous TB^4^	NoYes	2604 (97)74 (3)	7.5074.47	41/546411/140	110.14 (5.21–19.74)	<0.0001
Smoking (adults only; age>15 yrs, n = 1647)	NoYes	1495 (91)152 (9)	12.2627.64	38/30998/289	12.15 (1.00–4.60)	0.07
BCG vaccination^5^	NoYes	1182 (44)1482 (56)	9.379.29	23/245529/3120	11.00 (0.58–1.73)	0.99
*Findings at TB diagnosis of index cases:*						
Cavitation on CXR^6^	01–23–8	372 (15)1277 (53)760 (31)	11.227.749.94	9/80221/271315/1509	10.67 (0.31–1.48)0.83 (0.36–1.89)	0.61
Number of zones on CXR 7	0–34–56	1856 (72)616 (24)92 (3)	8.969.2215.81	35/390712/13013/190	11.03 (0.54–1.99)1.72 (0.53–5.60)	0.70
Cough duration of index case^8^ (weeks)	≤23–45–8>8	230 (9)1022 (38)662 (25)760 (28)	8.855.8511.8111.50	4/45213/222316/135518/1565	0.72 (0.23–2.20)1.38 (0.46–4.13)1.34 (0.46–3.97)	0.24

1 n = 221 missing;

2 based on n = 952;

3 n = 14 missing;

4 n = 1 missing;

5 n = 15 missing;

6 n = 270 missing;

7 n = 115 missing (from 8 households);

8 n = 5 missing (from 1 household); pyrs = person years; HR = hazard ratio; CI = confidence interval; CXR = chest radiograph; col = column; n**_max_** = maximum number.

**Table 4:** Univariable analysis of time to TB episode; hazard ratios, 95% CI and P-values (n_max_ = 2679) - Definite TB cases only (n = 40).

Variable	Description	n (col %)	Rate/1000 pyrs	TB cases/pyrs	Unadjusted HR(95% CI)	P-value
TST response^1^ (mm)	<10≥ 10	867 (35)1591 (65)	3.269.00	6/184030/3332	12.73 (1.14–6.56)	0.013
TST response 1 (mm)	<55–9.910–14.9≥ 15	548 (22)319 (13)688 (28)903 (37)	2.514.652.7713.76	3/19513/6454/144326/1889	11.73 (0.35–8.57)1.06 (0.24–4.73)5.31 (1.61–17.53)	0.0002
EC response^2^(SFC/Million PBMC)	<20≥ 20	337 (35)615 (65)	4.1111.30	3/73015/1327	12.75 (0.80–9.50)	0.076
EC response^2^(SFC/Million PBMC)	<32≥ 32	408 (43)544 (58)	3.4312.68	3/87515/1183	13.76 (1.09–13.00)	0.017
Gender	MaleFemale	1420 (53)1259 (47)	6.847.39	18/263022/2977	11.08 (0.58–2.01)	0.81
Age (years)	≤1516–29≥ 30	1031 (38)897 (33)751 (28)	1.8012.028.97	4/221622/1830141560	16.46 (2.23–18.76)4.86 (1.60–14.76)	0.0001
Proximity to index case at night^2^	Other houseSame houseSame roomSame bed	430 (16)1764 (66)213 (8)271 (10)	4.815.0411.221.30	4/83119/37665/44412/563	11.15 (0.39–3.39)2.54 (0.68–9.47)4.80 (1.54–14.88)	0.0001

1 n = 221 missing;

2 based on n = 952; pyrs = person years; HR = hazard ratio; CI = confidence interval; CXR = chest radiograph; col = column; n**_max_** = maximum number.

The incidence of TB for household contacts aged 5 years or less was 1.20/1000 pyrs (1 TB episode/834.9 pyrs) and increased with age (p<0.0001). History of previous episode of TB was also associated with an increased hazard of TB (p<0.0001). However, when restricting the analysis to household members with a prior history of TB, there was no association of time since last episode on TB incidence (p = 0.40; data not shown), but the numbers of events in the various time categories were small.

The risk of TB clearly increased with closer proximity of the household member to the index case at night time (p = 0.002), as well as with the amount of time and activities shared with the index TB case at day time, including eating daily with index case (p = 0.007). Amongst contacts aged >15 years, being a smoker increased the risk of TB two-fold (HR: 2.15, 95%CI: 1.00–4.60). There was no effect of BCG vaccination or factors reflecting infectivity of the index case on the time to TB episode in household contacts.

After adjustment for age, sex and proximity to index case at night time, the adjusted hazard ratio [aHR] for developing TB in two years follow-up when having a TST response of 10 mm or greater was reduced to 1.55 (95%CI: 0.74–3.24) (**Table 5**). The effect was stronger for a TST response greater or equal to 15 mm when compared to a response less than 5 mm (aHR: 2.56, 95%CI: 0.88–7.41). Household members with an ELISPOT response of 32 SFC/10^6^ PBMC or greater had nearly a two-fold increased rate of developing TB in follow-up compared to those with a response <32 (aHR: 1.98; 95%CI: 0.77–5.08). When restricting to definite TB cases only, the adjusted hazard ratio for active TB in contacts with positive TST response was similar to the analysis using all TB cases (aHR:1.73, 95%CI: 0.71–4.23), while it remained high for individuals with an ELISPOT response ≥32 SFC/10^6^ PBMC compared to those with a response <32 (aHR:3.61, 95%CI: 1.03–12.65, p = 0.02). An analysis taking into account the potential within household clustering using a Poisson random effects regression gave very similar results (data not shown).

**Table 5:** Multivariable analysis of time to TB episode (all TB cases and definite TB cases only); unadjusted and adjusted hazard ratios, 95% CI and P-values, separately for TST and EC responses.

Variable		n	Unadjusted HR	Adjusted HR^1^(95% CI)	P-value
*All TB cases (n = 52)*					
TST response (mm)	<10≥ 10	2458	12.37	11.55 (0.74–3.24)	0.23
TST response (mm)	<55–910–14≥ 15	2458	12.181.404.92	11.27 (0.34–4.82)0.73 (0.21–2.56)2.56 (0.88–7.41)	0.005
EC response(SFC/million PBMC)	<32≥ 32	951	12.13	11.98 (0.77–5.08)	0.14
*Definite TB cases (n = 40)*					
TST response (mm)	<10≥ 10	2458	12.73	11.73 (0.71–4.23)	0.20
TST response (mm)	<55–910–14≥ 15	2458	11.731.065.31	10.94 (0.19–4.72)0.52 (0.11–2.36)2.58 (0.76–8.74)	0.003
EC response(SFC/million PBMC)	<32≥ 32	951	13.77	13.61 (1.03–12.65)	0.02

1 adjusted for sex, age group, proximity to index case.

HR = hazard ratio; CI = confidence interval.

**Figure 2:**
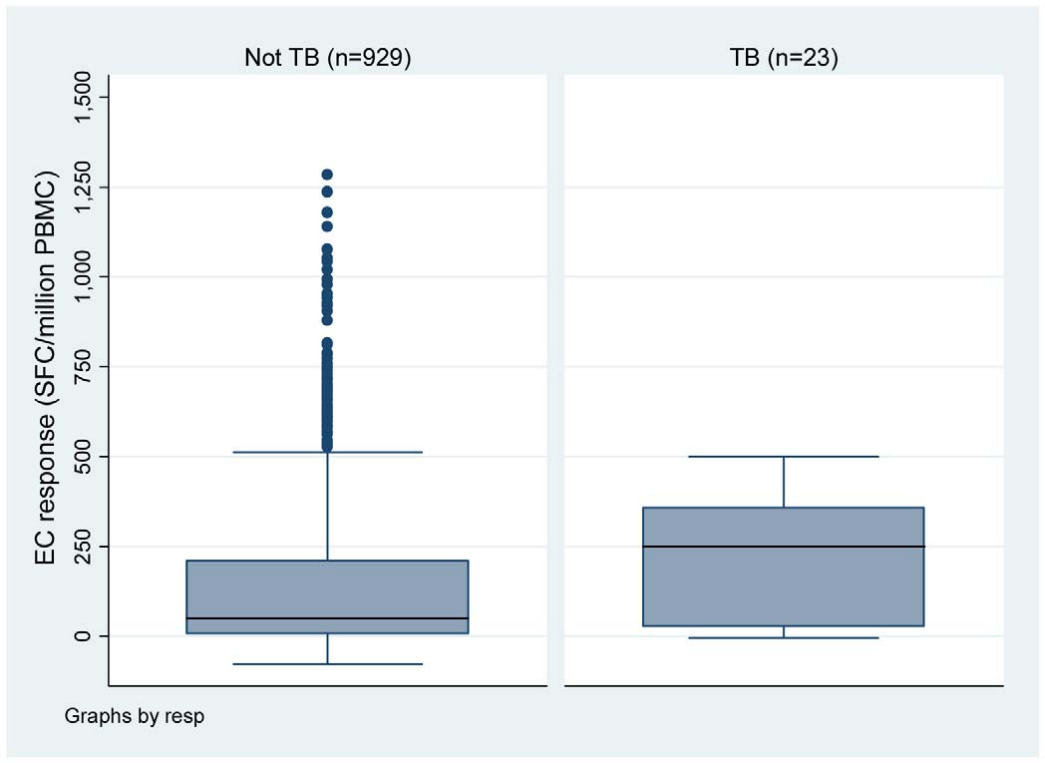
Box plots of EC ELISPOT response (SFC/million PBMC) at baseline by TB status in follow-up.

Quantitatively, the median ELISPOT response at baseline in household members who developed TB in follow-up was 250 *vs.* 50 SFC/10^6^ PBMC in those who did not develop TB (Wilcoxon rank-sum test, p = 0.02) (**Fig. 2**). Among household members with a positive ELISPOT response (≥32 SFC/10^6^ PBMC - n = 554), those with active TB in follow-up had a higher median response than those without TB (312.5 versus 185 SFC/10^6^ PBMC, respectively; p = 0.06).

A total of n = 893 household members had data on both TST and ELISPOT responses (**Table 6**). Those who had a positive response to both TST and ELISPOT had higher rate of TB in two years follow-up (14,74/1000 pyrs), compared to those positive on either test (11.15/1000 pyrs), although 95%CIs are wide and overlap. Individuals with both TST and ELISPOT negative response had a rate of TB of 9.85/1000 pyrs. However, households where the ELISPOT test was performed (n = 186) were similar to those where no ELISPOT test was carried out (n = 20), based on household size (p = 0.57), age (p = 0.57) and sex (p = 0.20) of the index case.

**Table 6:** Incidence rates by TST and EC response (restricted to those with both TST and EC response data, n = 893).

Description	n (col %)	TB cases/pyrs	Rate per 1000 pyrs	95% CI
TST <10 and EC<32TST ≥10 and EC<32TST <10 and EC≥32TST ≥10 and EC≥32	187 (21)193 (22)77 (9)436 (49)	4/4062/4061/17014/950	9.854.935.9014.74	3.79 – 26.251.23 – 19.700.83 – 41.878.73 – 24.89
TST≥10 or EC≥32	706 (79)	17/152	11.15	6.93 – 17.93
TST <10TST ≥10	264 (30)629 (70)	5/57616/1356	8.6911.80	3.62 – 20.877.23 – 19.27
EC<32EC≥32	380 (43)513 (57)	6/81115/1119	7.3913.40	3.32 – 16.458.08 – 22.23

CI = confidence interval; pyrs = person years.

## Discussion

In this prospective study of healthy household contacts of TB patients in Senegal, we found that the presence of either a positive TST or a positive EC ELISPOT response at baseline was associated with a two-fold increased hazard of time to active TB disease over a two year follow-up period compared to those who were Mantoux or ELISPOT negative. When adjusting on age, sex and proximity to case, all factors independently associated with development of TB in contacts of TB cases [15], the hazard of developing TB was 1.5 greater when TST response was >10 mm and 2.6 greater when TST response was >15 mm. Using the most stringent threshold, the time to TB episode in subjects with positive ELISPOT response at baseline remained about two-fold higher than in those with a negative response. Although not statistically significant, both estimates of the hazard ratios are comparable and in the same direction, suggesting that increased exposure to mycobacterial antigens is associated with increased risk of development of TB disease within the next two years. Moreover, there was a quantitative relation between the level of ELISPOT response and the risk of development of TB within two years follow-up. Remarkably, when restricting to definite TB cases only, the potential prognostic effect of a positive ELISPOT response appeared quite strong, with a nearly four-fold increased risk of active TB amongst household contacts.

A major limitation of our study was, however, the inability to ensure Isoniazid Preventive Therapy (IPT) for all child contacts≤5 years of age. As is the case with most low income countries, IPT is underused in Senegal. For example, WHO data [16] indicate that, in 2008, no HIV-infected patients had started IPT in Senegal, and the Senegal National TB Control Programme report states that, in 2008, only 1476 children in the whole country received IPT, for a detection of 11591 new smear positive TB cases. This reflects the limited use of IPT in the country, and dialogue has been initiated with the National TB Control Programme to stress the importance of following international guidelines. We hope our study will provide evidence to improve the existing situation and encourage wider use of IPT in child contacts of infectious TB cases.

Few prospective studies have been conducted so far to investigate the use of IGRAs as a marker of development of TB disease given infection [8]. Our estimates of the hazard ratio for time to TB episode incidence for TST or EC ELISPOT status at baseline are consistent with those observed in a cohort study conducted in a neighbouring country, The Gambia, in which the adjusted hazard ratio of developing TB in contacts was 1.8 (0.8–4.1) with the TST and 1.8 (0.8–4.2) with a similar in-house ELISPOT assay using similar threshold [13]. In a cohort of child contacts of infectious TB cases in Turkey, a positive response using commercial IGRA (T-SPOT.TB, Oxford Immunotec, Oxford, UK) was associated with a three-fold increase of risk of TB within two years follow-up [17], although the risk estimates were only adjusted on INH prophylaxis. Of note, the point estimates of risks were similar for TST and IGRA responses. Lastly, in a study in Germany using another commercial IGRA (QuantiFERON-TB Gold In-Tube, Cellestis, Carnegie, Victoria, Australia), the risk of developing TB was better predicted by a positive IGRA response in contacts who had a positive TST response (>5 mm), but this effect disappeared if the 10 mm threshold was used [18]. In all these studies, the risk of development of TB according to IGRA response was not adjusted on age, sex and proximity to TB cases. In this, our estimates can be considered more robust, and less liable to bias due to confounding factors.

Individuals with both TST and EC positive responses had a slightly higher incidence rate of TB (14.74/1000 pyrs) than individuals positive with either test alone (11.15/1000 pyrs), although 95%CI overlap. The high rate of TB in individuals with concordant negative TST and EC responses could be driven by concurrent HIV infection [19] that may affect the performance of the ELISPOT [12]. Individuals with discordant responses had the lowest rates of TB. In subjects with TST+/ELISPOT- responses, this could be due to a false positive TST, reflecting former sensitization by either BCG or environmental mycobacteria. This could also indicate a genuine difference between TST and IGRA in their ability to detect remote - and probably cleared - infection vs. recent, persisting TB infection [20]. The reason for TST-/ELISPOT+ response discordance is unclear, but could be related to variations around the respective thresholds, since both tests are continuous measures [20], [21].

The validation of IGRAs to detect LTBI suffers from the lack of gold standard, so active TB disease has often been used as a proxy marker for LTBI [4]. Using this approach, IGRAs have been shown to be as sensitive as, and more specific than, TST for detecting LTBI [5], [9]. Given this, it may be surprising that in our study a combination of both positive TST and positive ELISPOT appears just slightly better at predicting TB than a positive TST or a positive ELISPOT response alone, and that subjects with discordant responses to both tests had the lowest rate of developing TB. This could be understood if we consider that the detection of an adaptative immune response towards mycobacterial antigens is only a *footprint* of a former contact of the immune system with these organisms, which may not necessarily reflect the presence of living mycobacteria [21]. In this respect, TST and ELISPOT are probably not equivalent in what they measure. It has recently been suggested that T-cells responding to the RD antigens after 24 h stimulation are predominantly CD4 T-cells of an effector memory phenotype, consistent with having recently encountered antigens *in vivo*, while TST reflects the mobilization of a wider spectrum of memory T-cells that are long-lived and may even persist after clearance of live mycobacteria [21]. Recent data suggest that IGRA responses amongst contacts of TB cases are highly variable over time, and that a continued exposure to *M.tuberculosis* is necessary to maintain a high IGRA response [22], [23]. It has been hypothesized that, as individuals who have been *recently* exposed to TB offer a vigorous T-cell response to active bacterial replication [24], strong increases in IGRA response after recent exposure might predict progression to active disease [8], [25]. Our findings support this hypothesis, since the magnitude of the ELISPOT response was 5-fold higher in contacts who developed TB compared to those who did not develop TB, and the significant difference in performance of the ELISPOT compared to the TST was only seen in *definite* TB cases. This is in line with recent findings of a similar study in Columbia, a country of medium TB prevalence, where the rate of development of TB was highest in household contacts with high IFN-γ response to CFP-10 at baseline [26].

Current cut-off values for commercially available IGRA assays are set at relatively low levels in order to maximize the detection of individuals with *M. tuberculosis* infection who might benefit from chemoprophylaxis, especially amongst contacts of TB cases [27], [28]. In a setting where TB is endemic and households are large and likely to contain co-prevalent TB cases, it would be extremely difficult to determine the exact time of TB exposure (where an IGRA assay is likely to perform the best). In this situation there may be bias towards the longer lived TST response which outweighs the benefit of the increased specificity of the more transient IGRA response. Our findings indicate that a combination of the TST and ELISPOT might be best predictive of TB infection in household contacts of TB patients, and that the level of IFN-γ response to specific *M.tuberculosis* antigens may have prognostic value for the development of TB disease in this high-risk group [25], [26]. They confirm the need for extensive investigations of contacts of infectious TB cases when diagnosed, so as to identify individuals who may best benefit from chemoprophylaxis [29], [30] This should be conducted within the context of a full risk assessment that takes into consideration both individual characteristics and the intensity of exposure. However, given the highly dynamic nature of the IGRA response over time [23], [31], that probably reflects the variety of the underlying latent TB infection phenotypes [32], further prospective studies are needed to monitor conversion and reversion of IGRAs amongst contacts of infectious TB cases and their association with later development of TB. This would help defining a commonly acceptable threshold for definition of LTBI and the determination of a second cut-off that could be used to identify those most at risk of development of TB, who may therefore benefit most from preventive therapy.

## Supporting Information

Box S1 Certainty grading of diagnostic of TB in the Household Cohort Study - Senegal.
(0.03 MB DOC)
