# Correction: Evaluation of the Prognostic Value of IFN-γ Release Assay and Tuberculin Skin Test in Household Contacts of Infectious Tuberculosis Cases in Senegal

**DOI:** 10.1371/annotation/6aa24f81-7f3a-4163-b8bc-731c6112fcf7

**Published:** 2010-12-02

**Authors:** Christian Lienhardt, Katherine Fielding, Abdoul A. Hane, Aliou Niang, Cheikh T. Ndao, Farba Karam, Helen Fletcher, Fatou Mbow, Jules-François Gomis, Raymond Diadhiou, Maxime Toupane, Tandakha Dieye, Souleymane Mboup

The 10th author's name is misspelled. The correct name is Raymond Diadhiou. 

