# Supplementary material for: Evaluation of the Prognostic Value of IFN-γ Release Assay and Tuberculin Skin Test in Household Contacts of Infectious Tuberculosis Cases in Senegal
Source: PLoS One. 2010 May 6;5(5):e10508. doi: 10.1371/journal.pone.0010508 (PMC2865542; doi:10.1371/journal.pone.0010508)
Supplement: Box S1 — Certainty grading of diagnostic of TB in the Household Cohort Study - Senegal. (0.03 MB DOC) [file pone.0010508.s001.doc]

**Box S1. Certainty grading of diagnostic of TB in the Household Cohort Study- Senegal**

|  | **Adults** | **Children** |
| --- | --- | --- |
| *possible* | - sputum smears negative for AFB and clinical signs suggestive of TB *and/or* chest X-ray suggestive of TB (cavitation, miliary, ≥ 2 zones involved on CXR report)  *or*  *-* single “scanty” specimen on smear microscopy (less than 10 bacilli per 100 fields)  *or*  - signs and symptoms consistent with a suspicion of EPTB but no culture can be obtained | Any child presenting with any of the following:  - CXR *suggestive* of TB  *or*  - TST ≥15 mm if BCG scar present or TST ≥10 mm if BCG scar absent  *or*  - proven recent TST conversion and clinical signs and symptoms suggestive of TB (low weight for age or wasting, fever, persistent cough (> 2 weeks), cervical adenopathy, hepato-splenomegaly) |
| *probable* | - at least 1 sputum specimen positive for AFB on microscopy (except if there is only a single scanty microscopy positive specimen)  *And* chest X-ray suggestive of TB (cavitation, miliary, ≥ 2 zones involved on CXR report)  *or*  - signs and symptoms consistent with a suspicion of EPTB but no culture can be obtained and specific signs of EPTB on X-Ray or echography. | Any child with possible TB who had, in addition to the above:  - CXR with features *characteristics* of pulmonary TB (hilar or paratracheal adenopathy, mediastinal adenopathy, miliary TB)  *or*  - single scanty AFB result on microscopy (sputum smear or gastric aspirate) |
| *definite* | - at least two sputum smear positive for AFB  *or*  - one culture positive for *M. tuberculosis* | - at least one AFB positive result on microscopy (sputum smear or gastric aspirate), excluding a single scanty AFB result.  *or*  - one culture from any body tissue, fluids or secretion positive for *M.tuberculosis* |

AFB : Acid-Fast Bacilli

CXR: Chest X-Ray

EPTB: Extra-Pulmonary Tuberculosis
